# Cytotoxicity of Plumbagin, Rapanone and 12 other naturally occurring Quinones from Kenyan Flora towards human carcinoma cells

**DOI:** 10.1186/s40360-016-0104-7

**Published:** 2016-12-21

**Authors:** Victor Kuete, Leonidah K. Omosa, Viviane R. Sipowo Tala, Jacob O. Midiwo, Armelle T. Mbaveng, Sauda Swaleh, Oğuzhan Karaosmanoğlu, Hülya Sivas

**Affiliations:** 1 Department of Biochemistry, Faculty of Science, University of Dschang, Dschang, Cameroon; 2 Department of Chemistry, School of Physical Sciences, University of Nairobi, Nairobi, Kenya; 3 Faculty of Health Sciences, Université des Montagnes, Bangangte, Cameroon; 4 Department of Biology, Science Faculty, Anadolu University, Eskişehir, Turkey

**Keywords:** Carcinoma, cytotoxicity, Mode of action, Plumbagin, Quinones, Rapanone

## Abstract

**Background:**

Cancer is a major public health concern globally and chemotherapy remains the principal mode of the treatment of various malignant diseases.

**Methods:**

This study was designed to investigate the cytotoxicity of 14 naturally occurring quinones including; 3 anthraquinones, 1 naphthoquinone and 10 benzoquinones against 6 human carcinoma cell lines and normal CRL2120 fibroblasts. The neutral red uptake (NR) assay was used to evaluate the cytotoxicity of the compounds, whilst caspase-Glo assay was used to detect caspases activation. Cell cycle and mitochondrial membrane potential (MMP) were all analyzed via flow cytometry meanwhile levels of reactive oxygen species (ROS) were measured by spectrophotometry.

**Results:**

Anthraquinone: emodin (**2**), naphthoquinone: plumbagin (**4**), and benzoquinones: rapanone (**9**), 2,5-dihydroxy-3-pentadecyl-2,5-cyclohexadiene-1,4-dione (**10**), 5-*O*-methylembelin (**11**), 1,2,4,5-tetraacetate-3-methyl-6-(14-nonadecenyl)-cyclohexadi-2,5-diene (**13**), as well as doxorubicin displayed interesting activities with IC_50_ values below 100 μM in the six tested cancer cell lines. The IC_50_ values ranged from 37.57 μM (towards breast adenocarcinoma MCF-7 cells) to 99.31 μM (towards small cell lung cancer A549 cells) for **2**, from 0.06 μM (MCF-7 cells) to 1.14 μM (A549 cells) for **4**, from 2.27 μM (mesothelioma SPC212 cells) to 46.62 μM (colorectal adenocarcinoma DLD-1 cells) for **9**, from 8.39 μM (SPC212 cells) to 48.35 μM (hepatocarinoma HepG2 cells) for **10**, from 22.57 μM (MCF-7 cells) to 61.28 μM (HepG2 cells) for **11**, from 9.25 μM (MCF-7 cells) to 47.53 μM (A549 cells) for **13**, and from 0.07 μM (SPC212 cells) to 1.01 μM (A549 cells) for doxorubicin. Compounds **4** and **9** induced apoptosis in MCF-7 cells mediated by increased ROS production and MMP loss, respectively.

**Conclusion:**

The tested natural products and mostly **2**, **4**, **9**, **10**, **11** and **13** are potential cytotoxic compounds that deserve more investigations towards developing novel antiproliferative drugs against human carcinoma.

## Background

Cancer is a major public health problem globally killing about 3500 million people and representing 2–3% of the annual deaths [1]. Due to limited resources and other pressing public health problems, including communicable diseases such as acquired immune deficiency syndrome (AIDS), malaria, and tuberculosis, cancer continues to receive low public health priority in Africa, despite the growing burden of the disease [2]. Chemotherapy remains the principal mode of the treatment of various malignant diseases. In recent years, the search of antineoplastic compounds of natural origin has become more and more important. Many investigations are being carried out to identify new drugs or to find new lead structures from the flora of Africa to develop novel therapeutic agents for the treatment of human diseases such as cancer [3]. In our continous quest of lead molecules to fight cancer, we designed the present study to investigate the cytotoxicity of 14 quinones including 3 anthraquinones, one naphthoquinone and 10 benzoquinones, previously isolated from African medicinal plants. The study was extended to the study of the mode of action of the most active compounds including; plumbagin (**4**; a naphthoquinone) and rapanone (**9**; a benzoquinone). Quinones are secondary metabolites isolated principally from plants and having an aromatic di-one or di-ketone systems. Naturally occurring quinones are widely distributed and include benzoquinones, naphthoquinones, anthraquinones and polyquinones [4]. They exhibit numerous biological activities such as; neurological, antibacterial, antiplasmodial, antioxidant, trypanocidal, antitumor, antiviral activities [4, 5]. Amongst plant secondary metabolites, quinones comprise the second largest class of anticancer agents [6]. Some quinones isolated from African medicinal plants previously displayed anticancer activities. They include sargaquinoic acid isolated from *Sargassum heterophyllum* [7], 2-acetylfuro-1,4-naphthoquinone isolated from *Newbouldia laevis* [8], and anthraquinones such as damnacanthal, damnacanthol, 3-hydroxy-2-hydroxymethyl anthraquinone and schimperiquinone B obtained from *Pentas schimperi* [9].

## Methods

### Chemicals

**Fig. 1:**
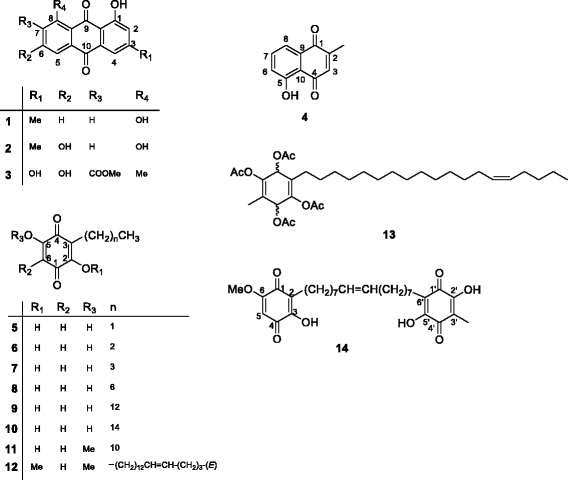
Chemical structures of the tested compounds. Chrysophanol (**1**), emodin (**2**), 3,6,8-trihydroxy-1-methylanthraquinone-2-carboxylic acid methyl ester (**3**), a naphthaquinone; 5-hydroxy-2-methyl-1,4-naphthalenedione or plumbagin (**4**), benzoquinones; 2,5-dihydroxy-3-ethyl-2,5-cyclohexadiene-1,4-dione (**5**), 2,5-dihydroxy-3-propyl-2,5-cyclohexadiene-1,4-dione (**6**), 2,5-dihydroxy-3-butyl-2,5-cyclohexadiene-1,4-dione (**7**), 2,5-dihydroxy-3-heptyl-2,5-cyclohexadiene-1,4-dione (**8**), 2,5-dihydroxy-3-tridecyl-2,5-cyclohexadiene-1,4-dione or rapanone (**9**), 2,5-dihydroxy-3-pentadecyl-2,5-cyclohexadiene-1,4-dione (**10**), 2-hydroxy-5-methoxy-3-undecyl-1,4-benzoquinone, 5-*O*-methylembelin (**11**), 2,5 dimethoxy-6-(14-nonadecenyl)-1,4-benzoquinone (**12**), 1,2,4,5-tetraacetate-3-methyl-6-(14-nonadecenyl)-cyclohexadi-2,5-diene (**13**), ardisiaquinone B (**14**)

The quinones (Fig. 1) used in this study were obtained from the chemical bank of the natural products research laboratory of the Chemistry Department, University of Nairobi, Kenya. Their isolation and identification were previously reported from the following plants: *Rumex dentatus*, *R. abyssinicus*, *R. usambarensis*, *R. bequaertii*, *R. ruwenzoriensis*, *R. crispus*; *Plumbago zeylanica*, *Myrsine Africana*, *Maesa lanceolata*, *Rapanea melanphloes*, *Aloe saponaria* [10]. They include anthraquinones: chrysophanol (**1**), emodin (**2**), 3,6,8-trihydroxy-1-methylanthraquinone-2-carboxylic acid methyl ester (**3**), a naphthaquinone: 5-hydroxy-2-methyl-1,4-naphthalenedione or plumbagin (**4**), benzoquinones; 2,5-dihydroxy-3-ethyl-2,5-cyclohexadiene-1,4-dione (**5**), 2,5-dihydroxy-3-propyl-2,5-cyclohexadiene-1,4-dione (**6**), 2,5-dihydroxy-3-butyl-2,5-cyclohexadiene-1,4-dione (**7**), 2,5-dihydroxy-3-heptyl-2,5-cyclohexadiene-1,4-dione (**8**), 2,5-dihydroxy-3-tridecyl-2,5-cyclohexadiene-1,4-dione or rapanone (**9**), 2,5-dihydroxy-3-pentadecyl-2,5-cyclohexadiene-1,4-dione (**10**), 2-hydroxy-5-methoxy-3-undecyl-1,4-benzoquinone or 5-*O*-methylembelin (**11**), 2,5 dimethoxy-6-(14-nonadecenyl)-1,4-benzoquinone (**12**), 1,2,4,5-tetraacetate-3-methyl-6-(14-nonadecenyl)-cyclohexadi-2,5-diene (**13**) and ardisiaquinone B (**14**) [10]. Doxorubicin 98.0% was purchased from Sigma-Aldrich (Munich, Germany) and used as reference drug.

### Cell lines and culture

Six human cancer cell lines and one normal cell line were used in this study. They included A549 human non-small cell lung cancer (NSCLC) cell line, obtained from the Institute for Fermentation, Osaka (IFO, Japan) and provided by Prof. Dr. Tansu Koparal (Anadolu University, Eskisehir, Turkey), SPC212 human mesothelioma cell line obtained from American Type Culture Collection (ATCC) and provided by Dr. Asuman Demiroğlu Zergeroğlu (Gebze Technical University, Kocaeli, Turkey), DLD-1 colorectal adenocarcinoma cell lines obtained from ATCC (CCL-221), Caco2 colorectal adenocarcinoma cells (ATCC, HTB-37) obtained from the ŞAP Institute of Turkey (Ankara), HepG2 hepatocarcinoma cells (ATCC, HB-8065) and MCF-7 breast adenocarcinoma cells (ATCC, HTB-22) were provided by Prof. Dr. Tansu Koparal (Anadolu University, Eskisehir, Turkey). The normal CRL2120 human skin fibroblasts were obtained from ATCC [CCD1094Sk (ATCC, CRL-2120)]. The cells were maintained as a monolayer in DMEM medium (Sigma-aldrich, Munich, Germany) medium supplemented with 10% fetal calf serum and 1% penicillin (100 U/mL)-streptomycin (100 μg/mL) in a humidified 5% CO_2_ atmosphere at 37 °C.

### Neutral red (NR) uptake assay

The cytotoxicity of samples was performed by NR uptake assay as previously described [11, 12]. This method is based on the ability of viable cells to incorporate and bind the supravital dye NR in the lysosomes. The procedure is cheaper and more sensitive than other cytotoxicity tests [13]. Samples were added in the culture medium so that dimethylsufoxide (DMSO) used prior for dilution did not exceed 0.1% final concentration. Briefly, cells were detached by treatment with 0.25% trypsin/EDTA (Invitrogen) and an aliquot of 1 × 10^4^ cells was placed in each well of a 96-well cell culture plate (Thermo Scientific, Germany) in a total volume of 200 μL. The cells were allowed to attach overnight and subsequently treated with different concentrations of the 14 compounds. Each of the studied samples was immediately added in varying concentrations in additional 100 μL of culture medium to obtain a total volume of 200 μL/well. After 72 h incubation in humidified 5% CO_2_ atmosphere at 37 °C, the medium was removed and 200 μL fresh medium containing 50 μg/mL NR was added to each well and incubation continued for an additional 3 h at 37 °C in 5% CO_2_ atmosphere. The dye medium was then removed and each well was then washed rapidly with 200 μL phosphate buffer saline (PBS) followed by addition of 200 μL of acetic acid-water-ethanol in water (1:49:50). The plates were kept for 15 min at room temperature to extract the dye and then shaken for a few minutes on a GFL 3012 shaker (Gesellschaft für Labortechnik mbH, Burgwedel, Germany). Absorbance was measured on ELx 808 Ultra Microplate Reader (Biotek) equipped with a 540 nm filter. Each assay was done at least three times, with three replicates each. The viability was evaluated based on a comparison with untreated cells. The IC_50_ values represent the sample’s concentrations required to inhibit 50% of cell proliferation and were calculated from a calibration curve by linear regression using Microsoft Excel [14].

### Flow cytometry for cell cycle analysis and detection of apoptotic cells

The cell-cycle analysis was performed by flow cytometry using BD cycletest^TM^ Plus DNA Kit Assay (BD Biosciences, San Jose, USA). The BD Cycletest™ Plus DNA kit provides a set of reagents for isolating and staining cell nuclei. Flow cytometric analysis of differentially stained cells is used to estimate the DNA index (DI) and cell-cycle phase distributions. Briefly, MCF-7 cells (3 mL, 1 × 10^5^ cells/mL) were seeded into each well of 6-well plates and allowed to attach for 24 h. The cells which were treated with ¼ × IC_50_, ½ × IC_50_ and IC_50_ concentrations of compounds **4**, **9** and the standard drug, doxorubicin were then grown in 6-well plates for 72 h. The untreated cells (control) were also included in the assay. They were further trypsinized and suspended in 1 mL PBS, then centrifuged at 400 g for 5 min at room temperature (RT). The cells were further processed according to the manufacturer protocol: addition of 250 μL of solution A (trypsin buffer), 10 min incubation at RT followed by the addition of 200 μL of solution B (trypsin inhibitor and RNAse buffer), 10 min incubation at RT followed by the addition of 200 μL of solution C (2–8 °C) (propidium iodide stain solution), 10 min on ice. The cells were further measured on a BD FACS Aria I Cell Sorter Flow Cytometer (Becton-Dickinson, Germany). For each sample, 10^4^ cells were counted. For PI excitation, an argon-ion laser emitting at 488 nm was used. Cytographs were analyzed using BD FACSDiva™ Flow Cytometry Software Version 6.1.2 (Becton-Dickinson).

### Caspase-Glo 3/7 and caspase-Glo 9 assay

Caspases activity in MCF-7 cells was detected using Caspase-Glo 3/7 and Caspase-Glo 9 Assay kits (Promega, Mannheim, Germany) as previously reported [15–17]. Cells were treated with compounds **4** and **9** at their 2 × IC_50_ and IC_50_ values with DMSO as solvent control for 6 h. Luminescence was measured using an BioTek Synergy™ HT multi-detection microplate reader. Caspase activity was expressed as percentage of the untreated control.

### Analysis of mitochondrial membrane potential (MMP)

The MMP was analyzed in MCF-7 cells by 5,5′,6,6′-tetrachloro-1,1′,3,3′-tetraethylbenzimidazolylcarbocyanine iodide) (JC-1; Biomol, Hamburg, Germany) staining as previously reported [15–17]. Cells (3 mL, 1 × 10^5^ cells/mL) treated for 72 h with different concentrations (¼ × IC_50_, ½ × IC_50_ and IC_50_) of compounds **4**, **9** and doxorubicin (drug control) or DMSO (solvent control) were incubated with JC-1 staining solution for 30 min according to the manufacturer’s protocol as reported in earlier. Subsequently, cells were measured in a BD FACS Aria I Cell Sorter Flow Cytometer (Becton-Dickinson, Germany). The JC-1 signal was measured at an excitation of 561 nm (150 mW) and detected using a 586/15 nm band-pass filter. The signal was analyzed at 640 nm excitation (40 mW) and detected using a 730/45 nm bandpass filter. Cytographs were analyzed using BD FACSDiva™ Flow Cytometry Software Version 6.1.2 (Becton-Dickinson). All experiments were performed at least in triplicate.

### Measurement of reactive oxygen species (ROS)

The 2′,7′-dichlorodihydrofluorescein diacetate (H_2_DCFH-DA) (Sigma-Aldrich) was used for the detection of ROS in MCF-7 cells treated with compounds **4**, **9** and doxorubicin (drug control) or DMSO (solvent control) using OxiSelect™ Intracellular ROS Assay Kit (Green Fluorescence) as recommended by the manufacturer, Cell Biolabs Inc. (San Diego, USA). This is a cell-based assay for measuring hydroxyl, peroxyl, or other reactive oxygen species activity within a cell. The assay employs the cell-permeable fluorogenic probe 2′,7′-dichlorodihydrofluorescin diacetate (DCFH-DA). DCFH-DA is diffused into cells and is deacetylated by cellular esterases to non-fluorescent 2′,7′-dichlorodihydrofluorescin (DCFH), which is rapidly oxidized to highly fluorescent 2′,7′-dichlorofluorescein (DCF) by ROS. Cells (1 × 10^4^ cells) were treated with samples at ¼ × IC_50_, ½ × IC_50_ and IC_50_ for 24 h. After addition of 100 μL 1× DCFH-DA/DMEM solution to cells and incubation at 37 °C for 30–60 min, the fluorescence was measured using SpectraMax® M5 Microplate Reader (Molecular Devices, Biberach, Germany) at 480/530 nm. All experiments were performed at least in triplicate.

## Results

The fourteen investigated compounds included three anthraquinones; chrysophanol C_15_H_10_O_4_ (**1**; *m*/*z*: 254.0579), emodin C_15_H_10_O_5_ (**2**; *m*/*z*: 270.0528), 3,6,8-trihydroxy-1-methylanthraquinone-2-carboxylic acid methyl ester C_17_H_12_O_7_ (**3**; *m*/*z*: 328.0583), one naphthaquinone; 5-hydroxy-2-methyl-1,4-naphthalenedione or plumbagin C_11_H_8_O_3_ (**4**; *m*/*z*: 188.0473), and benzoquinones; 2,5-dihydroxy-3-ethyl-2,5-cyclohexadiene-1,4-dione C_8_H_8_O_4_ (**5**; *m*/*z*: 168.0423), 2,5-dihydroxy-3-propyl-2,5-cyclohexadiene-1,4-dione C_9_H_10_O_4_ (**6**; *m*/*z*: 182.0579), 2,5-dihydroxy-3-butyl-2,5-cyclohexadiene-1,4-dione C_10_H_12_O_4_ (**7**; *m*/*z*: 196.0736), 2,5-dihydroxy-3-heptyl-2,5-cyclohexadiene-1,4-dione C_13_H_18_O_4_ (**8**; *m*/*z*: 238.1205), 2,5-dihydroxy-3-tridecyl-2,5-cyclohexadiene-1,4-dione or rapanone C_19_H_30_O_4_ (**9**; *m*/*z*: 332.4450), 2,5-dihydroxy-3-pentadecyl-2,5-cyclohexadiene-1,4-dione C_21_H_34_O_4_ (**10**; *m*/*z*: 350.2457), 2-hydroxy-5-methoxy-3-undecyl-1,4-benzoquinone, 5-*O*-methylembelin C_18_H_28_O_4_ (**11**; *m*/*z*: 308.1988), 2,5 dimethoxy-6-(14-nonadecenyl)-1,4-benzoquinone C_27_H_44_O_4_ (**12**; *m*/*z*: 432.3240), 1,2,4,5-tetraacetate-3-methyl-6-(14-nonadecenyl)-cyclohexadi-2,5-diene C_34_H_54_O_8_ (**13**; *m*/*z*: 590.3819), ardisiaquinone B C_30_H_40_O_8_ (**14**; *m*/*z*: 528.2723) [10]. These compounds are available in the Chemical bank of the Department of Chemistry, University of Nairobi, Kenya.

### Cytotoxicity

The cytotoxicity of the 14 quinones and doxorubicin was determined by the NR uptake assay and the recorded IC_50_ values are summarized in Table 1. The selectivity index was determined as the ratio of IC_50_ value in the CRL2120 normal fibroblast divided by the IC_50_ in the cancer cell line. Compounds **2**, **4**, **9**, **10**, **11** and **13** as well as doxorubicin displayed IC_50_ values below 100 μM in the six tested cancer cell lines. Compounds **3**, **5** and **12** were not active with IC_50_ values above 120 μM in all cancer cell lines meanwhile **1**, **6**, **7**, **8**, and **14** displayed selective activities. The recordable IC_50_ values were obtained in 1/6 tested cancer cell lines for **6**, 2/6 for **1**, 4/6 for **14** and 5/6 for **7** and **8**. Concerning the most active compounds, IC_50_ values ranged from 37.57 μM (towards breast adenocarcinoma MCF-7 cells) to 99.31 μM (towards small cell lung cancer A549 cells) for **2**, from 0.06 μM (towards MCF-7 cells) to 1.14 μM (against A549 cells) for **4**, from 2.27 μM (towards mesothelioma SPC212 cells) to 46.62 μM (against colorectal adenocarcinoma DLD-1 cells) for **9**, from 8.39 μM (towards SPC212 cells) to 48.35 μM (towards hepatocarinoma HepG2 cells) for **10**, from 22.57 μM (towards MCF-7 cells) to 61.28 μM (towards HepG2 cells) for **11**, from 9.25 μM (against MCF-7 cells) to 47.53 μM (against A549 cells) for **13**, and from 0.07 μM (towards SPC212 cells) to 1.01 μM (towards A549 cells) for doxorubicin. The six most active compounds (**2**, **4**, **9**, **10** and **13**) were generally less toxic towards normal CRL2120 fibroblast than carcinoma cells, and the obtained selectivity indexes were above 1.49, 1.96, 2.51, 2.91 and 59, respectively for **2**, **13**, **10**, **9** and **4**. Nonetheless, **11** as well as doxorubicin were in many cases slightly more toxic on normal CRL2120 fibroblast than on cancer cells (Table 1). Two compounds having the lowest IC_50_ values, namely **4** (below or around 1 μM in all the six cancer cell lines) and **9** (lowest IC_50_ value of 2.27 μM towards SPC212 cells) as well as doxorubicin were tested for the effects on cell cycle distribution, caspases activity, MMP breakdown and ROS production in MCF-7 cells.

**Table 1:** Cytotoxicity of tested compounds and doxorubicin towards cancer cell lines and normal cells as determined by the neutral *red* assay

Compounds	Cell lines, IC50 values in μM and selectivity index^a^ (in bracket)						
	A549	SPC212	DLD-1	Caco-2	MCF-7	HepG2	CRL2120
1	52.24 ± 5.51 (>3.01)	145.63 ± 10.63 (>1.08)	>157.48	>157.48	>157.48	>157.48	>157.48
2	66.30 ± 6.19 (>2.23)	99.31 ± 8.46 (>1.49)	77.28 ± 8.77 (>1.92)	73.63 ± 3.52 (>2.01)	37.57 ± 2.59 (>3.94)	71.7 ± 4.52 (>2.07)	>148.15
3	>121.95	>121.95	>121.95	>121.95	>121.95	>121.95	>121.95
4	**1.14** ± **0.02** (59.35)	**0.27** ± **0.01** (250.59)	**0.98** ± **0.11** (70.47)	**0.07** ± **0.01** (966.57)	**0.06** ± **0.01** (1127.67)	**1.01** ± **0.08** (66.99)	67.66 ± 6.49
5	>238.10	>238.10	>238.10	>238.10	>238.10	>238.10	95.51 ± 5.64
6	>222.22	>222.22	>222.22	>222.22	161.92 ± 11.37	>222.22	>222.22
7	68.62 ± 7.55 ((>2.97)	87.91 ± 5.66 (2.32)	>204.08	89.72 ± 8.80 (>2.27)	64.59 ± 13.42 (>3.16)	176.17 ± 28.5 (>1.16)	>204.08
8	107.52 ± 17.95 (1.09)	**8.05** ± **1.46** (1.09)	>168.07	63.93 ± 5.97 (2.27)	38.8 ± 3.33 (3.16)	94.22 ± 2.7 (1.16)	117.27 ± 1.22
9	27.35 ± 1.46 (3.48)	**2.27** ± **1.52** (41.96)	46.62 ± 5.38 (2.04)	22.95 ± 0.13 (4.15)	16.94 ± 4.65 (5.62)	32.69 ± 0.61 (2.91)	95.24 ± 6.65
10	43.32 ± 2.72 (>2.87)	**8.39** ± **0.48** (>14.81)	51.21 ± 5.54 (>2.43)	27.81 ± 2.03 (>4.47)	30.37 ± 8.64 (>4.09)	48.35 ± 3.73 (>2.57)	>124.24
11	50.26 ± 2.60 (0.88)	38.28 ± 3.23 (1.15)	45.37 ± 4.89 (0.97)	38.89 ± 2.35 (1.14)	22.56 ± 1.57 (1.96)	61.28 ± 5.53 (0.72)	44.20 ± 0.2
12	>129.45	>129.45	>129.45	>129.45	>129.45	>129.45	>129.45
13	47.53 ± 3.56 (1.96)	36.21 ± 3.08 (2.57)	25.09 ± 1.50 (3.72)	24.63 ± 2.71 (3.78)	**9.25** ± **0.16** (10.08)	18.17 ± 1.46 (5.13)	93.21 ± 1.17
14	21.68 ± 1.50 (3.29)	**3.14 ± 0.78** (22.72)	114.17 ± 3.68 (0.62)	>123.08	>123.08	114.60 ± 4.29 (0.62)	71.34 ± 5.23
Doxorubicin	**1.01** ± **0.19** (0.58)	**0.07** ± **0.01** (8.43)	**0.37** ± **0.05** (1.59)	**0.72** ± **0.13** (0.82)	**0.35** ± **0.05** (1.69)	**0.18** ± **0.03** (3.28)	**0.59** ± **0.01**

(^a^): The selectivity index was determined as the ratio of IC_50_ value in the CRL2120 normal fibroblasts divided by the IC_50_ in the cancer cell lines. Chrysophanol (**1**), emodin (**2**), 3,6,8-trihydroxy-1-methylanthraquinone-2-carboxylic acid methyl ester (**3**), plumbagin (**4**), 2,5-dihydroxy-3-ethyl-2,5-cyclohexadiene-1,4-dione (**5**), 2,5-dihydroxy-3-propyl-2,5-cyclohexadiene-1,4-dione (**6**), 2,5-dihydroxy-3-butyl-2,5-cyclohexadiene-1,4-dione (**7**), 2,5-dihydroxy-3-heptyl-2,5-cyclohexadiene-1,4-dione (**8**), 2,5-dihydroxy-3-tridecyl-2,5-cyclohexadiene-1,4-dione or rapanone (**9**), 2,5-dihydroxy-3-pentadecyl-2,5-cyclohexadiene-1,4-dione (**10**), 2-hydroxy-5-methoxy-3-undecyl-1,4-benzoquinone, 5-*O*-methylembelin (**11**), 2,5 dimethoxy-6-(14-nonadecenyl)-1,4-benzoquinone (**12**), 1,2,4,5-tetraacetate-3-methyl-6-(14-nonadecenyl)-cyclohexadi-2,5-diene (**13**), ardisiaquinone B (**14**). In bold: significant activity [3, 21, 22, 25]

### Cell cycle analysis and apoptosis

**Fig. 2:**
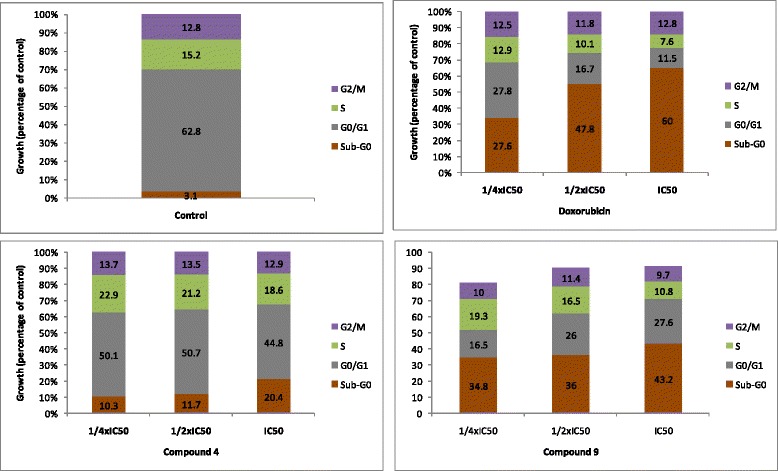
Effects of plumbagin (**4**), rapanone (**9**), and doxorubicin on cell cycle distribution in MCF-7 cells. IC_50_ values were 0.06 μM (**4**), 16.94 (**9**) and 0.35 μM (doxorubicin)

Naphthoquinone **4** and benzoquinone **9** were analyzed for their ability to alter the distribution of the cell cycle of MCF-7 breast cancer cells (Fig. 2). It was observed that the two compounds induced concentration-dependent cell cycle modifications with progressive increase of sub-G0/G1 phase cells. Compounds **4** and **9** induced cell cycle arrest between G0/G1 and S phases. MCF-7 cells treated with the compounds **4** and **9** progressively underwent apoptosis, with increase of sub-G0/G1 cells from 10.4% (¼ IC_50_) to 20.4% (IC_50_) for **4** and from 34.8% (¼ IC_50_) to 43.2% (IC_50_) for **9**. The positive control, doxorubicin also caused up to 60% sub-Go/G1 phase with IC_50_ treatment in comparison to only 3.1% in non-treated cells.

### Caspases activities

Upon treatment of MCF-7 cells with naphthoquinone **4** and benzoquinone **9** with equivalent (eq.) to the IC_50_ and 2-fold IC_50_ for 6 h, no modification of the activity of caspase 3/7 and caspase 9 was observed (data not shown).

### MMP breakdown

**Fig. 3:**
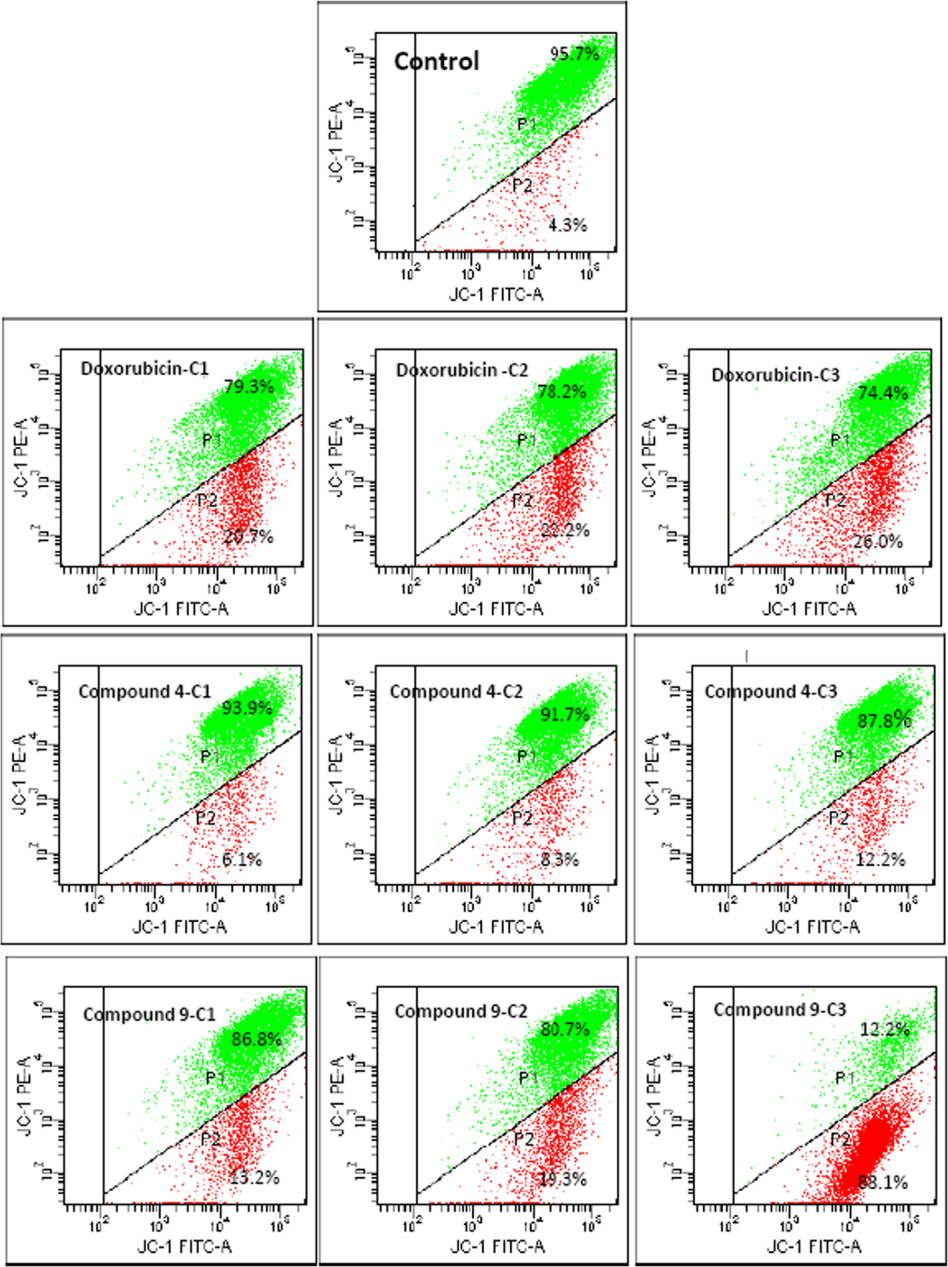
Effects of plumbagin (**4**), rapanone (**9**), and doxorubicin on MMP in MCF-7 cells for 72 h. IC_50_ values were 0.06 μM (**4**), 16.94 (**9**) and 0.35 μM (doxorubicin). Cells were treated with ¼ × IC_50_ (C1), ½ × IC_50_ (C2) and IC_50_ (C3) of each compound

Treatment of MCF-7 cells with compounds **4** and **9** with eq. to the 1/4 × IC_50_, 1/2 × IC_50_ and IC_50_ values for 72 h induced concentration-dependent depletion of MMP (Fig. 3). More pronounced effect was observed with **9** with up to 88.1% depletion of MMP at eq. to IC_50_ while **4** caused 12.2% MMP loss at IC_50_. In similar experimental condition, doxorubicin caused 26% loss of MMP meanwhile only 4.3% was observed with non-treated control.

### ROS production

**Fig. 4:**
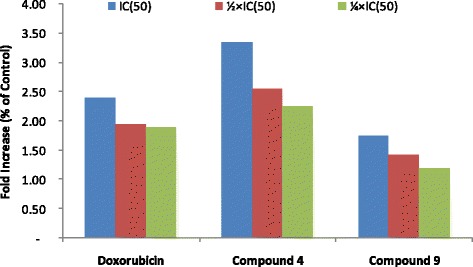
Induction of ROS in MCF-7 cells after treatment with plumbagin (**4**), rapanone (**9**), and doxorubicin for 24 h. IC_50_ values were 0.06 μM (**4**), 16.94 (**9**) and 0.35 μM (doxorubicin). Cells were treated with ¼ × IC_50_ (C1), ½ × IC_50_ (C2) and IC_50_ (C3) of each compound

After treatment of MCF-7 cells with naphthoquinone **4** and benzoquinone **9** at eq. to the 1/4 × IC_50_, 1/2 × IC_50_ and IC_50_ values for 24 h, the production of ROS in cells was analyzed (Fig. 4). Naphthoquinone **4** induced increased ROS levels of more than 3-fold (at IC_50_) as compared with non-treated cells meanwhile the increase was lesser (less than 2-fold) after treatment with benzoquinone **9**. In similar experimental condition doxorubicin also induced more than 2-fold increase in ROS production in MCF-7 cells at eq. to IC_50_.

## Discussion

Neoplastic diseases are one of the leading causes of mortality worldwide and the number of cancer cases are increasing regularly [1]. In general, leukemia cells are clinically more sensitive to chemotherapy than tumors [18, 19]. In the present study we focused on carcinoma cells involved in lung, colon, breast and liver cancers. In regards of the broad diversity of phytochemicals, the search of anticancer agents from plants represents an attractive strategy [20]. Molecules having IC_50_ values around or below 4 μg/mL or 10 μM [3, 21, 22] have been recognized as potential cytotoxic substances. IC_50_ values below 10 μM were observed with naphthoquinone **4** in all the six cancer cell lines. Interestingly, IC_50_ values below 1 μM were obtained with this compound in 4 of the 6 cancer cell lines, highlighting its good cytotoxic potential. In addition, the IC_50_ values obtained with **4** towards Caco-2 cells and MCF-7 cells were lower than that of the reference compound, doxorubicin. Other compounds such as **8**, **9** and **10** against SPC212 cells as well as **13** towards MCF-7 also displayed IC_50_ values below 10 μM, suggesting that they can be useful in the management of human carcinoma. Moreover, they were more toxic towards carcinoma cells than towards normal CRL2120 fibroblast (selectivity index > 1), indicating their good selectivity. The good activity obtained with naphthoquinone **4** is in accordance with previous studies. In fact, 2-acetylfuro-1,4-naphthoquinone previously displyed good cytotoxicity with IC_50_ values below 10 μM against a panel of cancer cell lines such as PF-382 leukemia T-cells, MiaPaCa-2 pancreatic cells, U87MG glioblastoma-astrocytoma cells, Colo-38 skin melanoma cells, HeLa and Caski cervical carcinoma cells [8]. Also, compound **4** is well known for its remarkable anticancer activities [6]. The present study therefore provides additional data on the anticancer potential of this compound and highlights the role of naphthoquinones as good cytotoxic compounds. The moderate cytotoxic effects of some anthraquinones such as damnacanthal, damnacanthol, 3-hydroxy-2-hydroxymethyl anthraquinone and schimperiquinone B on a panel of cancer cell lines was documented [9]. In the present study, the moderate to low activities obtained with anthraquinones **1**–**3** are also in accordance with such results. Induction of apoptosis is recognized as an efficient strategy for cancer chemotherapy and a useful indicator for cancer treatment and prevention. In the present study, it was found that compounds **4** and **9** induced apoptosis in MCF-7 cells (Fig. 2). Hence, further investigations of the mode of induction of apoptosis were performed. Caspases regulate apoptosis by cleaving cellular proteins at specific aspartate residues [23]. The activity of initiator caspase 9 and effector caspases 3/7 were investigated in MCF-7 cells treated with **4** and **9**. However, it was found that caspase-dependent cell death may not be one of the pathways of induction of apoptosis by **4** and **9** in MCF-7 cells. Loss of MMP is also classical evidence for apoptosis, occuring during the early stage of apoptosis before the cell morphology changes. The disruption of MMP was suggested to be very strong at percentages above 50%, and strong between 20 and 50% [3]; Up to 88.1% (at IC_50_) MMP depletion was obtained, when MCF-7 cells were treated with IC_50_ concentrations of **9**, suggesting that MMP depletion is involved in apoptotic pathway induced by this compound. ROS levels between 20 and 50% are considered as high [3]; More than 3-fold increase in ROS production was also obtained as results of treatment of MCF-7 with compound **4**. However, only 12.2% MMP depletion was obtained with this compound, suggesting that increase in ROS production the likely mode of apoptosis induced by naphthoquinone **4**. The tetraprenylquinone, sargaquinoic acid was shown to induce cycle arrest in MDA-MB-231 cells and apoptosis via increase in the activities of caspases 3, 6, 8, 9 and 13 [7]. However, it was demonstrated in this study that the related compound **9** induced apoptosis mediated by MMP loss but did not induced increase in the activity of caspases 3 and 9. Herein, it was also shown that compound **4** induced MMP loss in MCF-7 cells. Nonetheless, the induction was moderate. Compound **4** was also reported to induce apoptosis in PC-3 and DU145 cells, mediated by MMP loss [24]; this corroborates the results obtained in this work.

Regarding the structure-activity relationship, it appears that the naphthoquinone **4** was more potent in all six tested cancer cell lines than anthraquinones (**1**–**5**) and benzoquinone (**5**–**14**). Within anthraquinones, the substitution of hydroxyl (−OH) group in C8 (**1** and **2**) by a methyl group (**3**) significantly reduced the cytotoxic activity meanwhile the presence of − OH group in both C8 and C6 (**2**) seems to increase the activity. Consequently, IC_50_ values were obtained with **2** on all tested cancer cells lines and **1** on 2/6 (Table 1). Within benzoquinones, the degree of activity seems to increase with the size of the lateral chain in C2, the best effects being obtained between *n* = 10 (**11**) to *n* = 14 (**10**). However, compound **9** with *n* = 12 displayed better cytotoxic effects than **10** and **11** in all tested cancer cell lines (Table 1), most probably because of its higher lipophilicity.

## Conclusions

Finally, we demonstrated the cytotoxicity of naturally occuring quinones against human carcinoma cell lines. Naphthoquione **4** as well as anthraquinone **2** and benzoquinones **9**, **10**, **11**, and **13** displayed cytotoxic effects on all tested cancer cell lines. Compounds **4** and **9** induced apoptosis in MCF-7 cells mediated by increase ROS production and MMP loss repectively. The studied compounds and especially the most active ones deserve more investigations to develop novel cytotoxic drugs against cancers.

## Abbreviations

1: Chrysophanol
10: 2,5-dihydroxy-3-pentadecyl-2,5-cyclohexadiene-1,4-dione
11: 5-*O*-methylembelin
12: 2,5 dimethoxy-6-(14-nonadecenyl)-1,4-benzoquinone
13: 1,2,4,5-tetraacetate-3-methyl-6-(14-nonadecenyl)-cyclohexadi-2,5-diene
14: Ardisiaquinone B
2: Emodin
3: 3,6,8-trihydroxy-1-methylanthraquinone-2-carboxylic acid methyl ester
4: Plumbagin
5: 2,5-dihydroxy-3-ethyl-2,5-cyclohexadiene-1,4-dione
6: 2,5-dihydroxy-3-propyl-2,5-cyclohexadiene-1,4-dione
7: 2,5-dihydroxy-3-butyl-2,5-cyclohexadiene-1,4-dione
8: 2,5-dihydroxy-3-heptyl-2,5-cyclohexadiene-1,4-dione
9: Rapanone
DMSO: Dimethylsufoxide
H_2_DCF: 2′,7′-Dichlorodihydrofluorescein
H_2_DCFH-DA: 2′,7′-Dichlorodihydrofluorescein diacetate
MMP: Mitochondrial membrane potential
NR: Neutral red
PBS: Phosphate buffer saline
ROS: Reactive oxygen species
